# Efficient treatment of murine acute GvHD by in vitro expanded donor regulatory T cells

**DOI:** 10.1038/s41375-019-0625-3

**Published:** 2019-11-12

**Authors:** Christin Riegel, Tina J. Boeld, Kristina Doser, Elisabeth Huber, Petra Hoffmann, Matthias Edinger

**Affiliations:** 1 Department of Internal Medicine III, University Hospital Regensburg, Regensburg, Germany; 2 Institute of Pathology, University Regensburg, Regensburg, Germany; 3 Regensburg Center for Interventional Immunology, Regensburg, Germany; 4 Present Address: Comprehensive Cancer Center, Munich, Germany; 5 Present Address: Pathology Department, Red Cross Hospital, Munich, Germany

**Keywords:** Leukaemia, Allotransplantation, Peripheral tolerance, Bone marrow transplantation

## Abstract

Acute graft-versus-host disease (aGvHD) is a frequent complication after allogeneic bone marrow/stem cell transplantation (BMT/SCT) induced by co-transplanted alloreactive conventional donor T cells. We previously demonstrated that the adoptive transfer of donor CD4^+^CD25^+^Foxp3^+^ regulatory T cells (Treg) at the time of BMT prevents aGvHD in murine models. Yet, the therapeutic potential of donor Treg for the treatment of established aGvHD has not yet been studied in detail. We now used in vitro expanded phenotypically and functionally stable murine Treg to explore their therapeutic efficacy in haploidentical aGvHD models. Upon transfer donor Treg ameliorate clinical and histologic signs of aGvHD and significantly improve survival. They migrate to lymphoid as well as aGvHD target organs, predominantly the gastrointestinal tract, where they inhibit the proliferation of conventional T cells, reduce the influx of myeloid cells, and the accumulation of inflammatory cytokines. Successfully treated animals restore aGvHD-induced tissue damage in target organs and lymphoid tissues, thereby supporting lymphocyte reconstitution. The therapeutically applied Treg population survives long term without conversion into pathogenic effector T cells. These results demonstrate that donor Treg not only prevent aGvHD, but are also efficacious for the treatment of this life-threatening BMT complication.

## Introduction

Allogeneic BMT is a treatment option for a variety of malignant and nonmalignant hematologic diseases [1]. Mature T cells in the graft facilitate stem cell engraftment, enhance anti-infectious immunity and, most importantly, ensure the therapeutic graft-versus-leukemia (GVL) effect [2]. However, donor T cells can also recognize and attack host tissues thereby inducing acute graft-versus-host disease (aGvHD), the main cause of high morbidity and mortality in allogeneic BMT [3]. GvHD-induced tissue damage predominantly involves skin, liver, and the gastrointestinal (GI) tract [4]. Standard treatment for aGvHD are high-dose steroids with 50–65% response rates, while there is no established 2nd or 3rd-line therapy for nonresponders whose survival is dismal despite various salvage treatments [5].

CD4^+^CD25^+^Foxp3^+^ regulatory T cells (Treg) are thymus-derived suppressor cells that are pivotal for the prevention of autoimmunity by inhibiting the proliferation and differentiation of self-reactive T cells that escaped thymic deletion [6]. We and others showed that the adoptive transfer of such donor Treg with the BM graft does not induce aGvHD, but protects mice from otherwise lethal disease induced by alloreactive conventional donor T cells (Tconv) [7–10]. Importantly, donor Treg do not completely paralyze effector T cell function and therefore their GVL activity can be maintained [10–12]. Thus, donor Treg transfer for the prevention of aGvHD seems an attractive strategy for allo-HSCT and first proof-of-concept studies [13, 14] (and own unpublished results) confirmed their safety and efficacy.

Based on these encouraging results we explored for the first time in detail, whether donor Treg are also efficacious for the treatment of ongoing aGvHD after allogeneic BMT. To mimic the clinical situation, donor Treg were in vitro expanded and tested in haploidentical BMT models, where they profoundly ameliorated aGvHD, supported immune reconstitution and target organ regeneration and thereby rescued the majority of animals from otherwise lethal aGvHD.

## Material and methods

### Mice

Female BALB/c (H-2^d^), C57BL/6 (H-2^b^), and CB6F1 (H-2^bd^) mice were from Charles River Laboratories (Sulzbach, Germany), congenic BALB/c_Thy1.1 bred in-house. Donors were 6–9 weeks, recipients 9–10 weeks old at time of BMT. Mice were held under specific pathogen-free conditions and studies were approved by the Committee on Ethics of Animal Experiments at the Bavarian Government (Ref-No: 55.2-2532.1-18/11 and 55.2-2532-2-430).

### Cell isolation and sorting

Single cell suspensions from BM (femur and tibia) and spleen were prepared. Splenic CD25^+^ cells were enriched with anti-CD25-PE and anti-PE magnetic beads using MACS® (Miltenyi Biotec, Bergisch Gladbach, Germany), stained for CD4 and CD62L and sorted on a FACS-ARIA II® (BD Biosciences, Heidelberg, Germany) as CD4^+^ CD25^–^ Tconv and CD4^+^ CD25^high^CD62L^+^ Treg cells; purity was >98%.

For intestinal leukocyte isolation, small and large intestines were excised, Peyer’s patches removed, cut into 1–3 cm pieces, incubated twice for 15 min at 37 °C in HBSS/5mM EDTA/1mM DTT (Sigma-Aldrich, Munich, Germany) then vigorously shaken to isolate intraepithelial leukocytes (IEL). For lamina propria leukocytes (LPL), the fragments were transferred to DMEM with 0.13 U/ml Liberase^TM^ and 100 U/ml DNAse (Roche, Mannheim, Germany), mechanically dissociated, incubated at 37 °C for 30 min, further dissociated using GentleMACS® (Miltenyi Biotec), strained and then washed before Percoll centrifugation (75%/45%; IEL and small intestinal LPL) or magnetic separation using anti-CD45 beads (Miltenyi Biotec; colonic LPL).

### FACS

Staining was performed in PBS/2%FCS with anti-CD16/CD32 antibodies (BioLegend, Fell, Germany) to block FcR-binding and DAPI (Sigma-Aldrich, Munich, Germany) or Live/Dead (Thermo Fisher, Schwerte, Germany) to exclude dead cells. FACS was performed on a LSR II® (BD Biosciences) and data analyzed with FlowJo® (Treestar Inc., Ashland, OR). FACS-analysis/sorting antibodies are listed in Table S1.

### T cell expansion

Sorted CD4^+^CD25^high^CD62L^+^ Treg and CD4^+^CD25^–^ Tconv were cultured in DMEM (Gibco/Invitrogen, Darmstadt, Germany) with 10% FCS, 2 mM l-glutamine, 10 mM HEPES, 1% NEAA (PAN Biotech, Aidenbach, Germany), 50 U/ml penicillin, 50 µg/ml streptomycin, and 5 × 10^−5^ M 2-ME (Gibco/Invitrogen) in 96-U-well plates (1 × 10^4^/well) and stimulated with CD3/CD28-beads and rhIL-2 (Treg-Expansion Kit, Miltenyi Biotec, 4 and 2 beads/cell and Proleukin®, Chiron Amsterdam, Netherlands, 2000 and 100 U/ml for Treg and Tconv, respectively). Cultures were fed on day 4, restimulated on day 7 with 1 bead/cell after transfer into 24-well-plates (1 × 10^6^/ml/well), split on d11 and harvested on d14.

### Suppression assay

FACS-sorted, CFSE-labeled CD4^+^CD25^−^ responder T cells (Tresp, 5 × 10^4^/well) were seeded in 96-well plates with 1 × 10^5^ irradiated (30 Gy) autologous CD90-depleted splenocytes and 0.4 µg/ml anti-CD3ε in RPMI1640/5% FCS and supplements. Freshly isolated or in vitro expanded Treg or Tconv were added at indicated ratios, cultures were FACS-analyzed on day 3.

### GvHD model

CB6F1 recipients were randomly allocated to experimental groups on the day of BMT, irradiated (13Gy; split dose), and transplanted IV with 2.5 × 10^6^ BM cells with (GvHD) or without (BM control) 5 × 10^6^ splenocytes from BALB/c donors. On d11 post BMT, GvHD animals of the therapy group received 5 × 10^6^ in vitro expanded Treg (BALB/c or BALB/c_Thy1.1^+^). Recipients were monitored daily, body weight and GvHD symptoms assessed twice weekly by nonblinded investigators applying standardized scoring [15].

### Histopathology

Specimens obtained at indicated time points were fixed and embedded in paraffin, sections stained with hematoxylin/eosin (H/E) and GvHD scored by a blinded pathologist using established scoring systems [16]. Paneth cell numbers were quantified in H/E sections by an experienced pathologist (EH).

### Real-time quantitative PCR

Tissues were stored in RNAlater (Ambion, Oberursel, Germany); RNA was extracted from shredded tissue (RNeasy MiniKit, Qiagen, Hilden, Germany) and reverse-transcribed with M-MLV Reverse Transcriptase (Promega, Mannheim, Germany) after digestion of genomic DNA. Quantitative RT-PCR was done on a Mastercycler ep-gradient S (Eppendorf, Hamburg, Germany) using Brilliant III SYBR Green QPCR Master Mix (Agilent, Santa Clara, USA). Primers are listed in Table S2.

### Statistical analysis

Unless otherwise stated, results are shown as mean ± s.e.m. Statistics were calculated using GraphPad Prism 6 (GraphPad Software Inc., La Jolla, USA). For survival differences Kaplan–Meier analysis was performed and the log-rank test was used, for cellular subset differences a (paired) two-tailed Studentʼs *t* test or one/two-way ANOVA followed by Tukey’s multiple comparisons test, where appropriate. *P* values < 0.05 were considered significant (*p < 0.05; **p < 0.01; ***p < 0.001).

## Results

### In vitro expanded Treg ameliorate established aGvHD

**Fig. 1:**
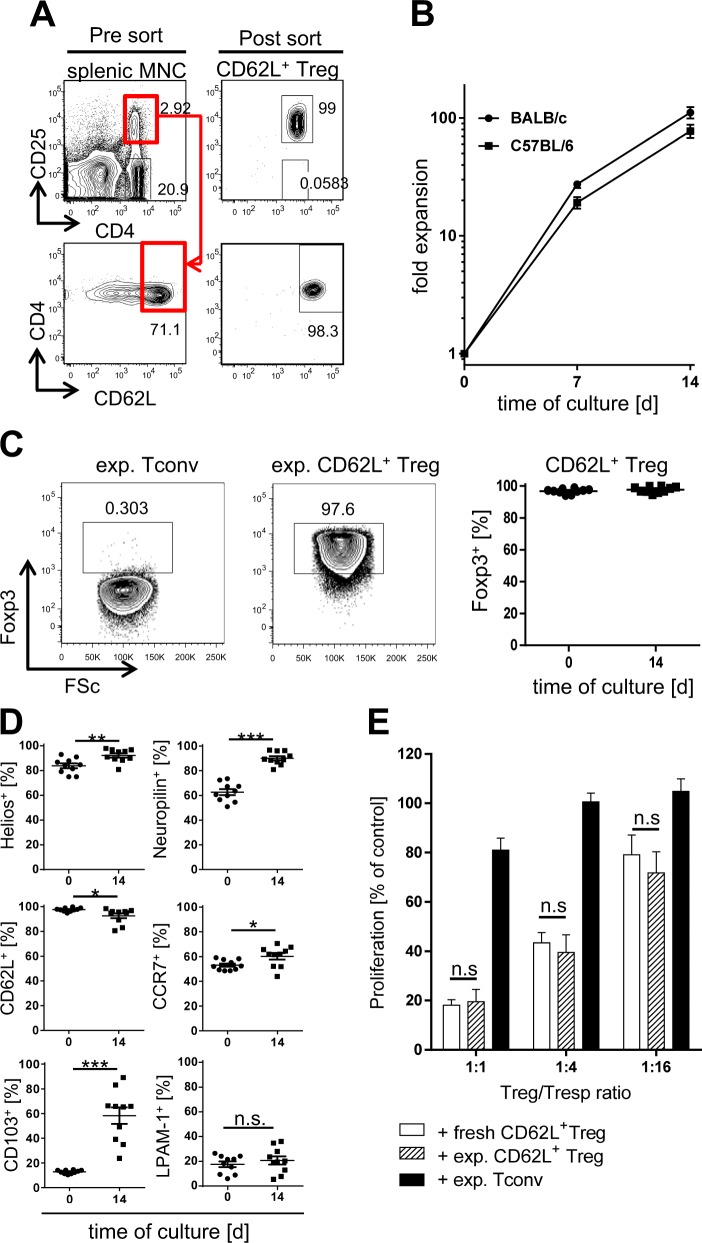
Murine CD62L^+^ Treg retain their phenotype and function during in vitro expansion and express gut homing receptors. CD4^+^CD25^high^CD62L^+^ Treg and CD4^+^CD25^−^ Tconv were FACS-sorted for in vitro expansion. **a** Gating strategy for sorting of CD4^+^CD25^high^CD62L^+^ Treg from BALB/c splenocytes and a representative example for sort purity according to CD4, CD25, and CD62L expression. **b** Expansion rates of FACS-purified BALB/c and C57BL/6 Treg (*n* = 20 independent cultures each). **c** Foxp3 expression of sorted Tconv and CD62L^+^ Treg after 14 days of in vitro culture. Representative FACS plots and summarized data of ten independent cultures. **d** Expression of Helios, Neuropilin, CD62L, CCR7, and the gut homing receptors CD103 and LPAM-1 on CD62L^+^ Treg before and after 14 days of in vitro culture (*n* = 10 independent cultures, *p < 0.05, **p < 0.01, ***p < 0.001). **e** Suppressive activity of Treg before and after in vitro expansion. Freshly isolated CFSE-labeled CD4^+^CD25^−^ Tresp cells were stimulated with anti-CD3ε in the presence of irradiated, autologous APC, and freshly sorted (combined data from five independent experiments) or in vitro expanded Treg (combined data from eight independent experiments) at the indicated ratios for 3 days. In vitro expanded Tconv served as negative control. Proliferation of Tresp cells was determined by FACS and is shown as % of proliferation observed in cultures with Tresp alone. All summarized data are shown as mean ± s.e.m

Due to their paucity in peripheral blood (PB), in vitro expansion of human Treg will be required prior to clinical application for aGvHD therapy. To mimic this situation, we isolated murine Treg by FACS-sorting splenic CD4^+^ CD25^high^CD62L^+^ cells (Fig. 1a) and cultured them with CD3/CD28-coated beads and rhIL-2. Restriction to the CD62L^+^ subpopulation enhances Treg purity for culture initiation [17] resulting in 96.6 ± 0.5% Foxp3 expression in sorted cells (*n* = 10) (Fig. 1a). Within 14 days, Treg of BALB/c and C57BL/6 origin expanded 111.4 ± 12.3 and 77.6 ± 10.0-fold (Fig. 1b), while maintaining Treg-specific Foxp3 and Helios expression and upregulating neuropilin-1 [18–21] (Figs. 1c, d and S1). CD62L and CCR7 expression also remained high, suggesting preserved homing capacity to secondary lymphoid organs (Fig. 1d) [22, 23]. Regarding gut homing receptor expression, the frequency of LPAM-1(α_4_β_7_ integrin)^+^ cells [24] remained stable during in vitro expansion, while CD103 (α_E_β_7_ integrin) [25] was significantly upregulated at variable levels (Fig. 1d). Homing receptors for other GvHD target organs, such as CCR9 [26], CCR4, and CLA [27, 28] were neither expressed by Treg ex vivo nor induced in culture (data not shown). Expanded Treg remained functional as they suppressed CD4^+^ Tconv proliferation in vitro as profoundly as freshly isolated Treg, while in vitro expanded FoxP3^−^ Tconv cells showed no relevant suppression (Fig. 1e).

**Fig. 2:**
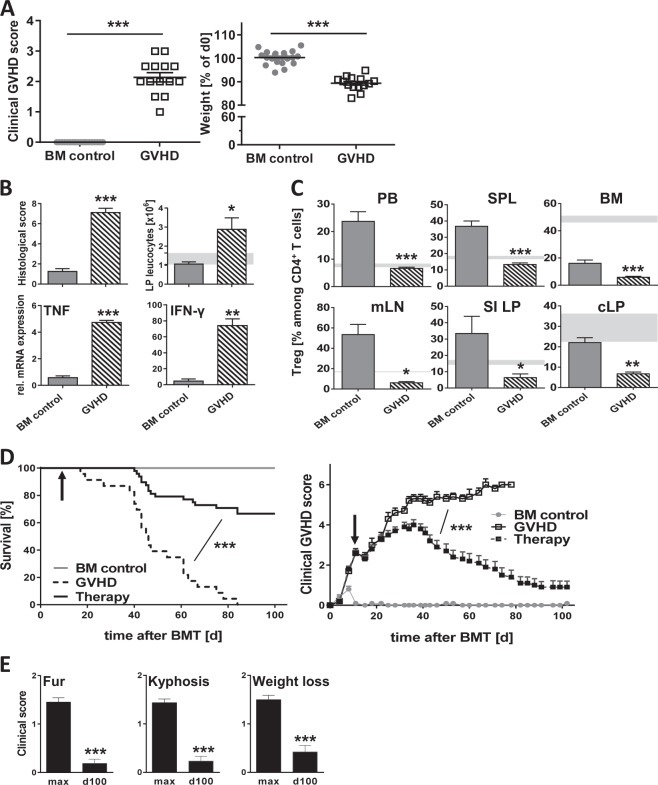
Therapeutic transfer of in vitro expanded donor Treg ameliorates established aGvHD and improves survival. **a**–**c** CB6F1 recipients were lethally irradiated, transplanted with 2.5 × 10^6^ BALB/c BM cells either alone (BM control; *n* = 20) or together with 5 × 10^6^ BALB/c splenocytes (GvHD; *n* = 14) and analyzed 11 days later (time point of Treg application). **a** Clinical GvHD score (left) and weight change (right) in relation to day 0. **b** Histopathological score (BM control: *n* = 6; GvHD: *n* = 9) and absolute leukocyte numbers in the LP of the colon (BM control: *n* = 5; GvHD: *n* = 6) as well as TNF and IFN-γ expression (*n* = 3 each); mRNA levels determined by qRT-PCR using HPRT as housekeeping gene and normalized to levels in nontransplanted mice. **c** Treg frequency among donor-derived CD4^+^ T cells in indicated tissues of GvHD (*n* = 3–12) and BM control mice (*n* = 4–20); shaded areas in **b** and **c** indicate respective levels in nontransplanted CB6F1 mice. **d** CB6F1 mice were lethally irradiated and transplanted with BALB/c BM alone (BM control; *n* = 18) or together with splenocytes (GvHD; *n* = 23) as stated above. On day 11 after BMT part of the GvHD animals received 5 × 10^6^ in vitro expanded BALB/c Treg (Therapy: *n* = 48). Survival (left) and clinical GvHD score (right) are shown. Treg application is indicated by the black arrow. **e** Peak and final clinical scores (maximum score = 2 per parameter) for fur texture (including alopecia), kyphosis, and weight loss of animals surviving for >90 days after Treg therapy (*n* = 32). Data in **a** are from 1 representative experiment out of 33, data in **b, c** are from 1–3 independent experiments and data in **d, e** are from 7 independent experiments. All summarized data are shown as mean ± s.e.m; *p < 0.05, **p < 0.01, ***p < 0.001

To test the therapeutic capacity of expanded Treg for the treatment of active aGvHD, we used a haploidentical parent→F1 (BALB/c→CB6F1) model. By day 11 post BMT, recipients of BM and splenocytes (“aGvHD mice”) showed weight loss and a significantly increased GvHD score as compared with mice receiving only BM (Fig. 2a and S2A–C). GI-GvHD was confirmed histologically and by cellular and molecular colon analysis revealing an increased leukocyte influx into the lamina propria, together with significantly augmented TNF and IFN-γ levels (Fig. 2b). Similar GvHD histopathology was observed in skin, liver, and small intestine (SI) (Fig. S2D). In accordance with findings in human aGvHD [29], we detected significantly lower frequencies of Treg among CD4^+^ T cells in PB, spleen, BM, and mLN as well as the LP of small and large intestine in aGvHD mice (Fig. 2c).

Next, we treated mice with established aGvHD with in vitro expanded BALB/c donor Treg on day 11 post BMT and monitored survival as well as clinical symptoms for the next 90 days. Nontreated GvHD mice succumbed to progressive disease at a median of 46 days and all animals died by day 85 post BMT (Fig. 2d), whereas BM control mice survived until day 100 without symptoms. aGvHD mice receiving donor Treg did not immediately respond, yet clinical improvement became obvious around day 23 after therapy (i.e., day 34 post BMT), when Treg-treated animals showed a significantly diminished GvHD score (3.8 ± 0.3 as compared with 5.2 ± 0.2 for nontreated aGvHD mice; *p* < 0.001). Importantly, symptoms further improved over time and 66.7% of Treg-treated animals survived the observation period of 100 day (Fig. 2d). Comparing peak and final scores for different clinical parameters in surviving animals revealed that Treg-treated mice indeed recovered from aGvHD-induced tissue damage (Fig. 2e).

To prove that therapeutic effects of donor Treg are not limited to this donor-recipient strain combination, the alternative parental strain (C57BL/6) was used as BM and Tconv ± Treg donor. This way, GvHD-relevant variables (e.g., MHC-disparity, irradiation dose, animal vendor, microbiome, and environmental conditions) were kept constant to focus on Treg-mediated effects. In this less aggressive aGVHD model (25% of nontreated GvHD animals survived), donor Treg treatment also significantly improved clinical GvHD symptoms, histopathologic changes, and overall survival (66.7% survival of Treg-treated animals; *p* < 0.01; Fig. S3),

### Adoptively transferred donor Treg migrate preferentially to the GI tract, persist long term, and remain Foxp3^+^

**Fig. 3:**
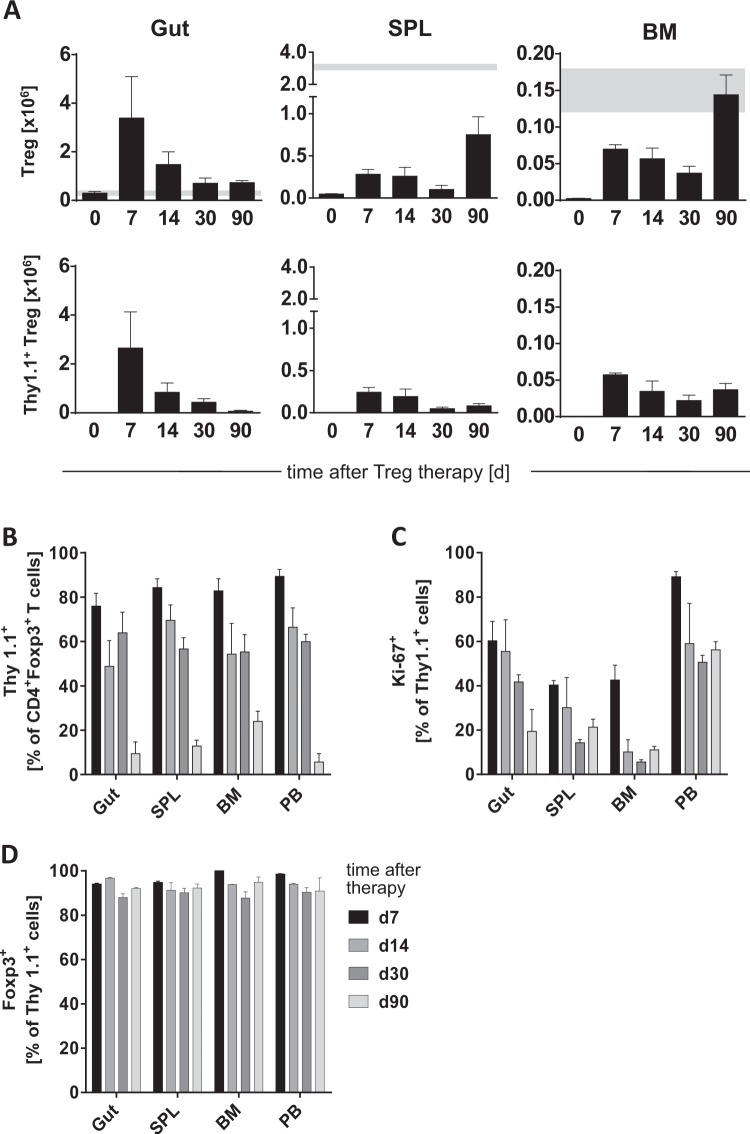
Therapeutically applied Treg predominantly migrate to the GI tract, persist long term, and retain a stable phenotype. CB6F1 recipients were irradiated and GvHD was induced by transplantation of WT (Thy1.2^+^) BALB/c BM and splenocytes as detailed in Fig. 2. On day 11 after BMT, animals received additional 5 × 10^6^ in vitro expanded congenic (Thy1.1^+^) BALB/c Treg. **a** Cell numbers of total CD4^+^Foxp3^+^ Treg (top) and Thy1.1^+^ therapeutically applied Treg (bottom) in indicated organs at indicated time points after Treg application (gut: *n* = 3 (pools of 5–6 mice each); spleen: *n* = 3–12/time point; BM: *n* = 3–12/time point). Shaded areas indicate Treg levels in respective organs of nontransplanted CB6F1 mice. **b** Relative proportion of therapeutically applied (Thy1.1^+^) among total Treg in indicated organs at indicated time points after Treg therapy (gut: *n* = 3 (pools of 5–6 mice each); spleen: *n* = 3–9/time point; BM and PB: *n* = 3–10/time point). **c** Proportion of Ki-67^+^ (proliferating) cells among therapeutically applied (Thy1.1^+^) Treg (gut: *n* = 3–7 (pools of 5–6 mice each/time point); spleen: *n* = 3–10/time point; BM and PB: *n* = 3–8/time point). **d** Foxp3 expression among therapeutically applied (Thy1.1^+^) Treg in indicated organs at indicated time points after injection (gut: *n* = 3 (pools of 5–6 mice each); spleen: *n* = 3–9/time point; BM and PB: *n* = 3–10/time point). Combined data are from three independent experiments and are shown as mean ± s.e.m

To analyse their migratory behavior and persistence, in vitro expanded Treg from congenic Thy1.1^+^ BALB/c mice were injected into CB6F1 recipients of BALB/c (Thy1.2^+^) BM and splenocytes on day 11 post BMT. Seven days post Treg therapy substantially increased Treg numbers were recovered from all examined organs (Fig. 3a, upper panels) and the vast majority (>70%) of them were derived from the therapeutically administered Treg population (Fig. 3a, lower panels; gating strategy illustrated in Fig. S4). Highest absolute numbers (3.4 ± 1.7 × 10^6^) were retrieved from the gut, representing >80% of all therapeutic Treg reisolated from the indicated organs and exceeding by far the physiological Treg numbers in the GI tract of nontransplanted mice (gray area in Fig. 3a, upper panel). Over time, Treg numbers in the gut declined, yet therapeutic Treg still constituted 9.44 ± 5.26% of the local pool 90 days after transfer (Fig. 3b). The comparatively modest increase in absolute Treg numbers in spleen and BM one with 4 weeks after therapy was also predominantly driven by therapeutically applied Treg. In contrast to the gut, however, Treg numbers further increased over time in both lymphoid organs, reaching their maximum not before day 90 after Treg application. With the amelioration of aGvHD this Treg reconstitution evolved from the BM graft (see below) while the proportion of Thy1.1^+^ (therapeutic) Treg decreased, but even at day 90 therapeutically applied Treg and their progeny still constituted 6, 13, and 24% of all Treg in PB, spleen, and BM, respectively (Fig. 3b) (9, 13, and 4.7% in the C57BL/6→CB6F1 model shown in Fig. S3C–D). Throughout the whole observation period therapeutic Treg remained proliferative (as determined by Ki-67 expression) with peak proliferation rates of up to 60% in the GI tract 1 week after transfer and still ranging from 10 to 20% by day 90 after injection in all analyzed organs. Interestingly, circulating therapeutic Treg maintained a high proportion of proliferating cells at all time points tested (Fig. 3c). Finally, we examined the lineage stability of administered Treg and found stable Foxp3 expression (>90% in all organs) even after 3 months (Fig. 3d).

### Treg therapy of active aGvHD reinvigorates lympho-/hematopoietic reconstitution

**Fig. 4:**
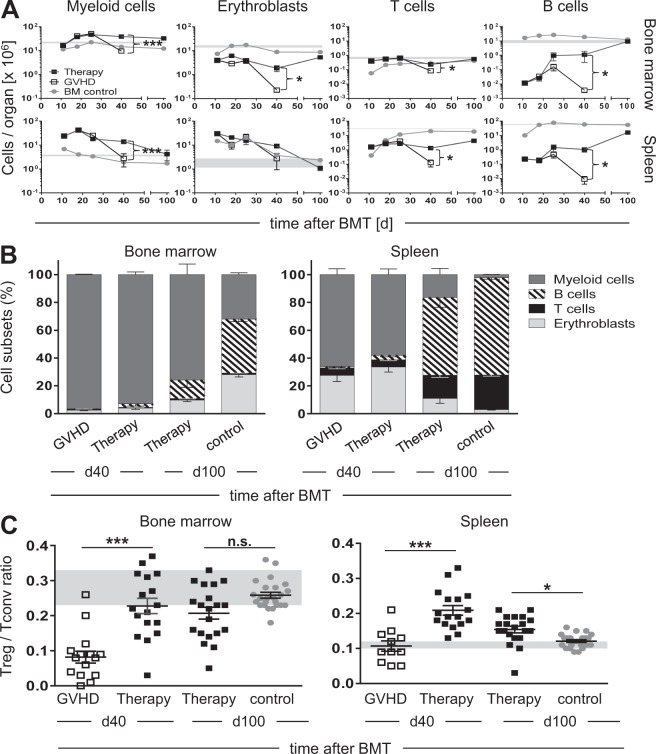
Treg therapy fosters lympho-/hematopoietic reconstitution. CB6F1 recipients were irradiated and transplanted as detailed in Fig. 2. On day 11 after BMT part of the aGvHD mice received expanded Treg (Therapy). At indicated time points mice from each group were sacrificed and cells were analyzed by FACS (BM control: *n* = 7–25/time point; GvHD: *n* = 10–17/time point; Therapy: *n* = 11–20/time point; combined data from more than three independent experiments). Absolute cell numbers (**a**) and relative proportions among MNC (**b**) of myeloid cells (CD11b^+^; 1st panel), erythroblasts (CD45^−^Ter119^+^; 2nd panel), T cells (TCRαβ^+^; 3rd panel) and B cells (CD19^+^, 4th panel) in BM (2 hind legs), and spleen are shown (GvHD day 40: *n* = 10; Therapy day 40: *n* = 17; Therapy day 100: *n* = 16; BM control day 100: *n* = 24; combined data from more than three independent experiments). **c** Treg/Tconv ratios in BM and spleen 40 and 100 days after allogeneic BMT (GvHD day 40: *n* = 16(BM)/11(spleen); Therapy day 40: *n* = 17; Therapy day 100: *n* = 20; BM control day 100: *n* = 24; combined data from more than three independent experiments). Data represent mean ± s.e.m (*p < 0.05, **p < 0.01, ***p < 0.001)

To examine the influence of Treg therapy on myeloid and lymphoid reconstitution, we serially analyzed BM and spleen. BMT recipients without aGvHD showed a rapid and full reconstitution of their myeloid compartment in the BM. Acute GvHD accelerated myelopoiesis as illustrated by increased absolute and relative numbers of myeloid cells (macrophages and predominantly neutrophils) in the BM and spleen early after BMT (Fig. 4a, 1st panel and Fig. 4b; gating strategy illustrated in Fig. S5).

Interestingly, all BMT recipients showed an initial accumulation of erythroid precursors in the spleen, indicating extramedullary erythropoiesis. BM erythropoiesis was rapidly restored in control mice and only modestly impaired in early aGvHD, but totally collapsed in final GvHD stages (Fig. 4a, 2nd panel). T cell reconstitution in BM and spleen of aGvHD-free mice was comparably slow and normal values were not reached before day 100. In comparison, T cell numbers in aGvHD were increased sevenfold in BM and fourfold in spleen on day 11 after BMT and consisted mainly of donor T cells that induced splenic fibrosis leading to dramatically reduced T cell numbers in end-stage aGvHD (Fig. 4a, 3rd panel). The most sensitive indicator of GvHD-induced lymphoid deterioration, however, was the B cell compartment in BM and spleen as control mice showed a rapid and full reconstitution of their B cell compartments, while aGvHD dramatically impaired B cell regeneration (Fig. 4a, 4th panel and Fig. 4b). Intriguingly, aGvHD mice treated with in vitro expanded donor Treg reconstituted their B cell compartment and normalized their myelo- and erythropoiesis in BM and spleen over time and showed a significantly improved T cell recovery in both compartments (Fig. 4a, b).

To further explore the influence of therapeutic Treg on alloreactive Tconv, we determined the ratio of these populations. In BM the Treg frequency was significantly diminished in end-stage aGvHD but restored to normal levels in Treg-treated animals by day 40 post BMT (Fig. 4c). In the spleen, the Treg frequency seemed normal in aGvHD mice (albeit on a low absolute level; see Fig. 4a), but rose above physiological levels upon Treg treatment to 21% on day 40 and remained elevated until day 100 (Fig. 4c).

### Therapeutic application of donor Treg dampens inflammation and promotes tissue regeneration in the GI tract

**Fig. 5:**
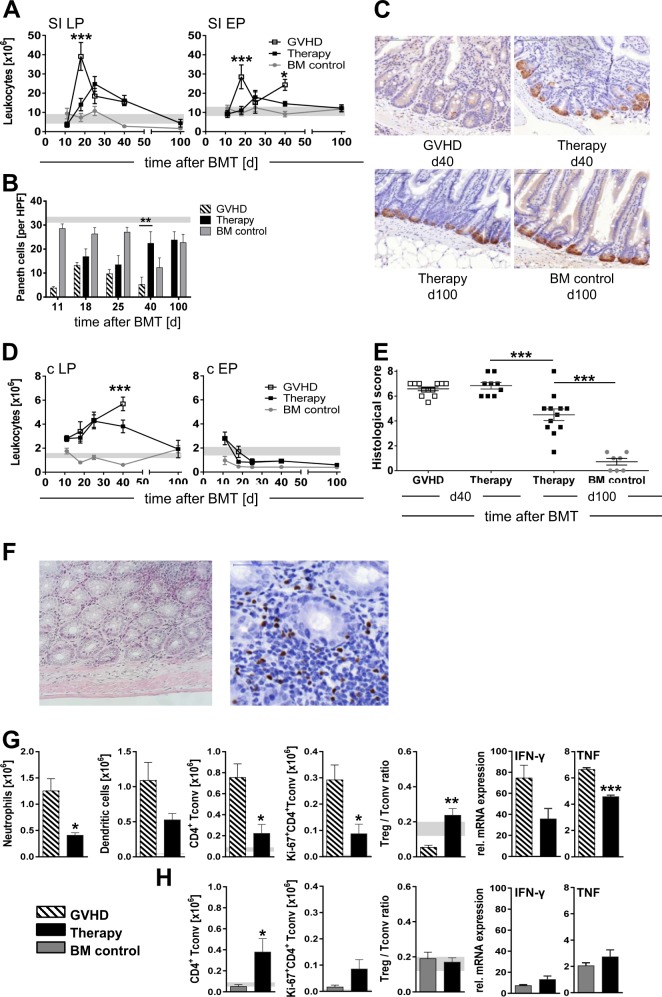
Therapeutic Treg dampen inflammation and support tissue regeneration in the GI tract. CB6F1 recipients were conditioned and transplanted with either BM cells alone (BM control; *n* = 12–24/time point) or with additional splenocytes (GvHD; *n* = 10–17/time point) as detailed in Fig. 2. On day 11 after BMT, part of the GvHD animals received donor-derived in vitro expanded Treg cells (Therapy; *n* = 10–20/time point). At the indicated time points mice were sacrificed and small intestine (SI) and colon were analyzed histopathologically, by FACS and by qRT-PCR. **a** Absolute numbers of CD45^+^ leukocytes in SI lamina propria (LP) and epithelium (EP). Presence and function of Paneth cells in the SI: **b** Paneth cells/high power field (HPF, magnification 40×, BM control: *n* = 6–7/time point; GvHD: 5–15/time point; therapy: *n* = 8–13/time point) and **c** lysozyme staining of representative SI specimens (magnification 40×). **d** Absolute cell numbers of leukocytes in colon LP and epithelium (BM control: *n* = 12–24/time point; GvHD: 10–17/time point; therapy: *n* = 10–20/time point) and **e** histopathological score of the colon at indicated time points post BMT (GvHD day 40: *n* = 12; therapy day 40: *n* = 9; therapy day 100: *n* = 12; BM control day 100: *n* = 7). **f** Representative histology after Treg therapy showing mostly normal colon architecture and an area of leukocyte infiltrates (left panel, H/E staining, magnification 200×) and the high frequency of Treg (dark brown/black) in such colonic infiltrates (right panel, staining for Foxp3, magnification 400×). Absolute cell numbers of indicated leukocyte subpopulations, Treg/Tconv ratios and TNF and IFN-γ expression (normalized to levels in nontransplanted animals) in the colon of aGvHD and Treg therapy animals at day 40 after BMT (**g**) and of Treg therapy and BM control animals at day 100 after BMT (**h**) (*n* = 3 for cytokines day 40; *n* = 4–8 for all other analyses on day 40 and day 100). Combined data from 1 (cytokines day 40) or 3–4 independent experiments are shown. Neutrophils are defined as CD45^+^CD11b^+^Gr-1^+^, DC as CD45^+^CD11c^+^. Proliferating CD4^+^TCRαβ^+^Foxp3^−^ Tconv are identified by Ki-67 expression. Gray-shaded areas indicate respective levels in nontransplanted CB6F1 mice. Summarized data are shown as mean ± s.e.m, (*p < 0.05, **p < 0.01, ***p < 0.001)

As affection of the GI tract is the main cause of morbidity and mortality in aGvHD we assessed the impact of Treg therapy on GI damage. By day 18 after BMT aGvHD mice—unlike control mice—showed a massive infiltration of leukocytes into the LP and epithelium of the SI (*p* < 0.001). This influx was significantly reduced and delayed in Treg-treated aGvHD mice with maximum cell numbers not reached before day 25 after BMT. By day 100, leukocyte numbers in both SI compartments of Treg-treated recipients were back to normal and comparable with those in aGvHD-free BM controls (Fig. 5a).

Paneth cells in the SI secrete antimicrobial compounds such as α-defensins and are pivotal for microbiome homeostasis. Recent clinical and experimental studies linked Paneth cell loss to aGvHD severity [30, 31]. In our experiments, BM control animals displayed a steady number of Paneth cells (23.39 ± 2.94/HPF) with only a transient drop on day 40 after BMT (Fig. 5b). Animals with aGvHD showed significantly reduced numbers of Paneth cells by day 11 (3.79 ± 0.60/HPF), followed by a transient low-level regeneration (13.2 ± 1.26/HPF) by day 18 and a nearly complete loss (5.28 ± 3.04/HPF) by day 40. In sharp contrast, aGvHD mice receiving Treg therapy on day 11 replenished their Paneth cells by day 40 and maintained comparable levels with BM control mice until day 100 (23.77 ± 3.46/HPF and 22.71 ± 3.46/HPF, respectively). Functional integrity of regenerated Paneth cells was confirmed by histochemical staining for lysozyme (Fig. 5c).

Histology also confirmed severe colon damage in aGvHD, showing dense mixed inflammatory infiltrates (lymphocytes and neutrophils), crypt cell apoptosis, deep mucosa ulcerations, and marked crypt distortion and loss (Fig. S6). By comparison, histopathologic changes were significantly reduced in successfully treated aGvHD mice by day 90 after therapy (Fig. 5e) with large areas of the colon showing normal crypt architecture with increased epithelial regeneration (Fig. 5f, left panel). In some areas, however, crypts were displaced by a lymphocytic infiltrate (Fig. 5f, left panel right upper corner) that displayed high Treg frequencies (Fig. 5f, right panel). These observations were confirmed by FACS (Fig. 5d) revealing a rapid and steadily increasing leukocyte influx into the LP in aGvHD that ceased 4 weeks after Treg treatment, the time point when clinical symptoms improved (see Fig. 2d). When analyzed in detail, the number of neutrophils, dendritic cells, and CD4^+^ Tconv infiltrating the colon LP of aGvHD mice at day 40 were decreased by 69, 50, and 71%, respectively, after Treg therapy (Fig. 5g). This was accompanied by a significant (70%; *p* < 0.05) decrease in Tconv proliferation as well as a significantly (*p* < 0.01) increased Treg/Tconv ratio. Similarly, elevated TNF and IFN-γ levels were considerably reduced 4 weeks after Treg therapy (Fig. 5g). By day 100, overall leukocyte numbers in the colon of Treg-treated mice (1.9 ± 0.7 × 10^6^/colon) were comparable with those of BM controls (1.9 ± 0.3 × 10^6^/colon; Fig. 5d). The number of Tconv, however, was still slightly elevated, as was their proliferative activity (Fig. 5h). Yet, due to the parallel increase in colonic Treg the Tconv/Treg ratios in Treg-treated aGvHD mice remained similar to those in control animals (0.17 ± 0.02 and 0.19 ± 0.04, respectively). In addition, TNF and IFN-γ levels had further declined by day 100 and were comparable with those observed in BM controls that had never experienced aGvHD.

Taken together, these results demonstrate that therapy of established aGvHD with in vitro expanded donor Treg ameliorates ongoing GvHD, halts inflammation, fosters immune reconstitution, and promotes tissue regeneration and thereby rescues animals from lethal aGvHD.

## Discussion

Donor Treg prevent aGvHD after allogeneic BMT as previously shown by us and others in various experimental models [7–9]. First clinical trials confirmed the safety and efficacy of donor Treg for the prevention of aGvHD [13, 14, 32]. Encouraged by these findings, we explored in detail whether donor Treg have therapeutic potential for the treatment of ongoing aGvHD. For this purpose a haploidential BMT model was used in which co-transplanted splenocytes induce severe aGvHD with involvement of the GI tract as revealed by clinical symptoms, histology, cellular, and molecular analyses. The adoptive transfer of in vitro expanded donor Treg 11d after disease establishment improved the aGvHD-associated deficiency in absolute and relative Treg numbers, improved clinical symptoms over time and rescued 66.7% aGvHD mice while untreated animals succumbed to progressive disease. Donor Treg did not elicit an immediate response, but animals gradually recovered from aGvHD tissue damage beginning ~2–3 weeks post Treg therapy. In early experiments by R. Korngold’s group therapeutic effects of donor Treg were observed in CD8-dependent minor histocompatibility antigen (miHA)-disparate BMT models, but they were ineffective in haploidentical models (B6→B6 × C3HF1) even when administered as early as day 4 post BMT [33]. Differences in Treg purity (Foxp3 was not yet discovered), the activation status (freshly isolated vs. in vitro expanded) and cell numbers (1 Mio vs. 5 Mio/animal in our study) may explain this discrepancy, as R. Negrin’s group showed that increased Treg numbers are required to control aGvHD [10]. Sufficiently high numbers of functional Foxp3^+^ Treg were generated by FACS-purification of the CD62L^+^ Treg subpopulation that ensured high purity and prevented outgrowth of contaminating non-Treg, as previously reported [17]. Polyclonal in vitro expansion was applied to mimic clinical scenarios where donor Treg expansion seems inevitable for the treatment of aGvHD due to their paucity in PB [34, 35]. Importantly, Foxp3 expression was not only maintained in vitro but also for more than 3 months after transfer in vivo, illustrating that the reported conversion of Treg into pathogenic effector cells under inflammatory or lymphopenic conditions [36, 37] is not a major issue in aGvHD. One week after their injection the majority of Treg resided within the GI tract where they represented ~80% of the local Treg pool. Similar frequencies were detected in BM, spleen, and PB and they remained at high levels for 4 weeks before they declined to ~10–25% by day 100. This percent reduction was not solely caused by a decline in absolute numbers of therapeutically transferred Treg, but also associated with lymphoid reconstitution and regeneration of BM-derived Treg in successfully treated GvHD mice (Fig. 3).

Impaired immune reconstitution due to Tconv-mediated destruction of primary and secondary lymphoid organs is a hallmark of aGvHD [38–41] and contributes to the frequent opportunistic infections in aGvHD patients [42]. The most sensitive indicator of aGvHD-induced lymphoid disturbance and regeneration after Treg therapy was the B cell compartment. While aGvHD-free BMT recipients rapidly restored their B cell pool, aGvHD resulted in its complete loss in the BM and periphery, as shown previously in experimental models [43] (and own unpublished results) and clinical studies [44]. Treg treatment of aGvHD fostered B cell regeneration in BM and spleen leading to almost normal cell numbers and distributions at the end of the observation period. We previously showed that in steady-state BM contains the highest frequency of Treg among CD4^+^ T cells in the body [7]. Importantly, this high Treg frequency is crucial for the protection of the hematopoietic stem cell niche [45] that seems disturbed in mice with aGvHD, but rapidly restored upon Treg treatment (Fig. 4). Thus, donor Treg therapy seems to ameliorate so-called “BM-GvHD” [43, 46] and protect the SC and the B cell precursor niche, as previously also observed in scurfy mice [47]. Whether this is a direct Treg effect or mediated indirectly through suppression of cytokine-secreting or cytotoxic Tconv is currently under investigation. Yet, donor Treg clearly inhibited Tconv proliferation in the BM as early as day 7 after transfer.

GI damage is the main cause of death in aGvHD and severe GI tissue destruction was observed at the time of Treg treatment. In the SI of untreated mice, an almost complete loss of Paneth cells occurred, a pathognomonic finding in murine and human aGvHD [30, 31]. Importantly, Paneth cells rapidly regenerated in Treg-treated aGvHD mice, reached normal numbers already by day 40 and were functionally intact as evidenced by lysozyme staining. This finding is astonishing and clearly illustrates the tissue regenerating capacity of donor Treg therapy. Loss of Paneth cell-secreted antimicrobial peptides (e.g., Reg3γ and α-defensins) has been shown to cause dysbiosis in aGvHD that perpetuates disease progression [48, 49]. Although not formally proven in our experiments, their regeneration seems to disrupt this vicious circle and to support the reestablishment of microbial homeostasis; In addition, Paneth cells are pivotal components of the epithelial stem cell niche [50] and Treg-mediated Paneth cell regeneration may thus protect epithelial stem cells, themselves targets of the GvH-reaction [51]. In combination, Tregs clearly support the restoration of the mucosal barrier function.

Tissue regeneration after Treg therapy was also observed in the colon, where histology, a high leukocyte and Tconv influx, elevated inflammatory cytokines and diminished Treg frequencies confirmed severe aGvHD before Treg treatment. As in SI cellular responses to Treg treatment were already detectable by day 40 with a significant increase in Treg, a concomitant reduction in Tconv numbers and proliferation, reduced DC and neutrophil infiltration [52], and decreased cytokine secretion. All parameters further stabilized and were close to GvHD-free controls (BMT w/o splenocyte transfer) by day 100. The sole difference was areas of increased lymphocytic infiltrates (within normal histology areas) that contained Treg in high frequencies. These findings suggest that aGvHD amelioration and the promotion of tissue regeneration by Treg is a continuous active process and not the result of donor Tconv ablation. The relevance of donor Treg persistence for long-term tolerance after BMT needs further clarification and is currently under investigation.

As shown here for aGvHD, donor Treg recently also proved effective for the treatment of chronic GvHD [53]. Despite these promising results, clinical translation remains a challenge [54]. Ritz and colleagues previously described that low-dose IL-2-treatment preferentially increases Treg and ameliorates cGvHD, at least in a substantial proportion of patients [55, 56]. Based on these reports, several groups initiated clinical trials to explore the adoptive transfer of Treg for cGvHD [57] (see also: www.tregeneration.eu), in parts combined with low-dose IL-2 therapy (J. Koreth, Dana Faber Cancer Institute, NCT01937468). Most groups use magnetic bead-based isolation technologies that ensure a 60–85% Treg enrichment [32, 35]. For aGvHD treatment, however, large cell numbers are presumably required that are only achievable through in vitro expansion, for which contaminations with Tconv must be omitted. We previously described in vitro expansion protocols using CD45RA^+^ naive Treg for culture initiation, which permits the generation of pure Treg products [58] without contamination with Tconv or memory-type Treg prone to lose FOXP3 expression upon repetitive stimulation [58–60]. Such protocols were now adapted to GMP standards (including GMP-approved FACS-sorting) for an ongoing phase I–II proof-of-concept study exploring the treatment of refractory aGvHD patients (EudraCT 2012-002685-12). In this trial, administered Treg cell numbers (10 Mio/kg body weight) closely match the human equivalent dose deducted from the mouse experiments as calculated by the model of Reagan-Shaw et al. [61]. Recently described alternative technologies suggest that high Treg purity and expansion rates are also achievable without FACS-sorting, which will further facilitate the clinical translation of this approach [14]. The data presented here strongly support the use of Treg as therapeutic agents for aGvHD as they reversed tissue damage, supported immune reconstitution, and improved survival in otherwise lethal aGvHD.

## Supplementary information

Supplemental material
