# Supplementary material for: Efficient treatment of murine acute GvHD by in vitro expanded donor regulatory T cells
Source: Leukemia. 2019 Nov 12;34(3):895–908. doi: 10.1038/s41375-019-0625-3 (PMC7214258; doi:10.1038/s41375-019-0625-3)
Supplement: Supplementary file 1 — Supplemental material [file 41375_2019_625_MOESM1_ESM.pdf]

Supplemental Methods

Immunohistochemistry: Immunohistochemical staining of 1 µm sections was done after deparaffinization and rehydration. Endogenous peroxidases were blocked by peroxidase-blocking-solution (Dako, Jena, Germany). For lysozyme staining, sections were incubated with a polyclonal rabbit-anti-lysozyme antiserum (Dako, Jena, Germany) followed by a peroxidase-labelled anti-rabbit secondary reagent (N-Histofine® Simple Stain MAX PO, anti-rabbit; Medac, Wedel, Germany). For Foxp3 staining, a monoclonal rat-anti-Foxp3 antibody (clone: FJK-16s, eBioscience, Frankfurt, Germany) was used, followed by a peroxidase-labelled anti-rat secondary reagent (N-Histofine® Simple Stain MAX PO, anti-rat; Medac, Wedel, Germany). All incubations were done for 45 min followed by two washing steps. Staining was developed by adding a 3,3'-diaminobenzidine (DAB) chromogen/substrate reagent (Dako, Jena, Germany) for 10 min. Sections were again washed thoroughly, counter-stained with hematoxylin/eosin and mounted with non-aqueous mounting medium (Entellan; Merck, Darmstadt, Germany).

Table S1: Antibodies used for flow cytometry

Antibody	Clone	Fluorochrome	Source
**CD3**	145-2C11	FITC, APC, APC-Cy7	eBioscience
**CD4**	RM4-5	FITC, PE, PerCP, APC, PE-Cy7, PB	BD Biosciences, eBioscience, BioLegend
**CD8α**	53-6.7	PerCP, APC	BD Biosciences
**CD11b**	M1/70	PE, PE-Cy7, PerCP-Cy5.5	BD Biosciences
**CD11c**	N418	eFluor450, PerCP	eBioscience
**CD11c**	HL3	PerCP-Cy5.5	BD Biosciences
**CD16/32**	93	Purified	BioLegend
**CD19**	1D3	FITC, PE-Cy7	BD Biosciences
**CD19**	6D5	PE, APC-Cy7	BioLegend
**CD25**	PC61	PE, APC, PE-Cy7	BD Biosciences, BioLegend
**CD45**	30F-11	FITC, PE	BD Biosciences
**CD62L**	MEL-14	APC, PE-Cy7	BD Biosciences, eBioscience
**CD90.1**	OX-7	PerCP, PE-Cy7	BD Biosciences, eBioscience
**CD90.2**	53-2.1	PB	BioLegend
**CD103**	2E7	FITC, APC	eBioscience
**CD197/CCR7**	4B12	PE-Cy7	eBioscience
**CD304**	REA380	PE, APC	Miltenyi Biotec
**Foxp3**	FJK-16s	FITC, PE, APC, PB	eBioscience
**Gr-1**	RB6-8C5	APC	BD Biosciences
**H2K^b**	AF6-88.5	FITC, PE, Biotin	BioLegend
**Helios**	22F6	PE	BioLegend
**Ki-67**	SolA15	eFluor660	eBioscience
**LPAM-1**	DATK32	PE	BD Biosciences
**TCRβ**	H57-597	PE, APC, PE-Cy7	BD Biosciences
**TCRβ**	REA318	APC	Miltenyi Biotec
**TER-119**	TER-119	APC	BioLegend

Table S2: Primers used for real-time quantitative PCR

Primer	F/R	Sequence (5‘ → 3‘)
**IFN-γ**	F	TCAAGTGGCATAGATGTGGAAGAA
**IFN-γ**	R	TGGCTCTGCAGGATTTTCATG
**TNF**	F	CATCTTCTCAAAATTCGAGTGACAA
**TNF**	R	TGGGAGTAGACAAGGTACAACCC
**HPRT**	F	CTCATGGACTGATTATGGACAGGAC
**HPRT**	R	GCAGGTCAGCAAAGAACTTATAGCC

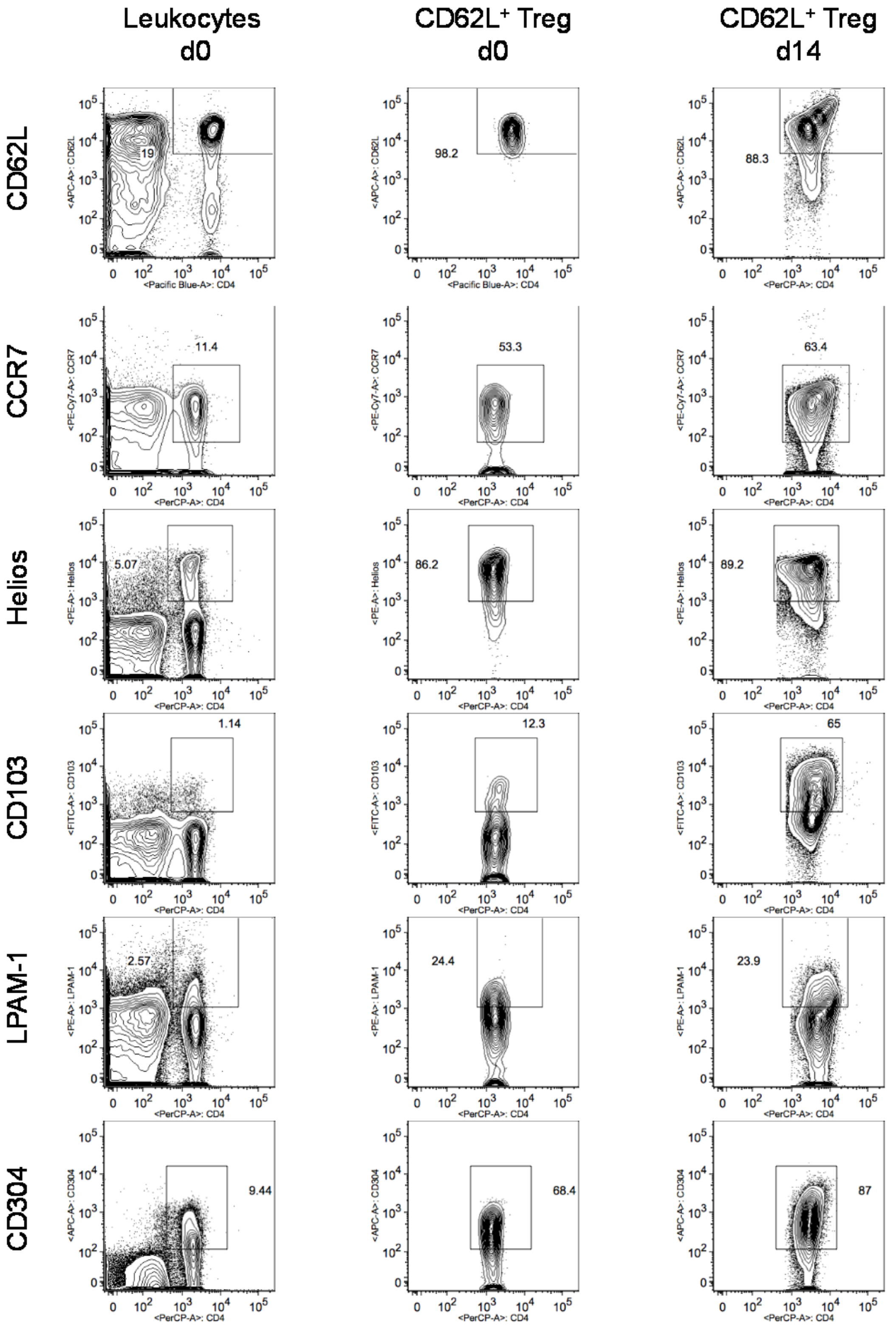

Figure S1 (related to Fig. 1D): Marker expression profiles of $CD62L^+$ Treg cells before and after 14d of *in vitro* culture. Gates for each particular marker were set according to expression profiles of freshly isolated $CD4^+$ splenocytes after dead cell and doublet exclusion (left column). For CD62L, gates were set to comprise the $CD62L^{high}$ subset only. Treg cells on d0 (middle column) were identified as $CD4^+CD25^{high}CD62L^{high}$ among splenic MNC. Analysis of Treg on d14 of *in vitro* culture (right column) was performed on all live $CD4^+$ T cells after doublet exclusion.

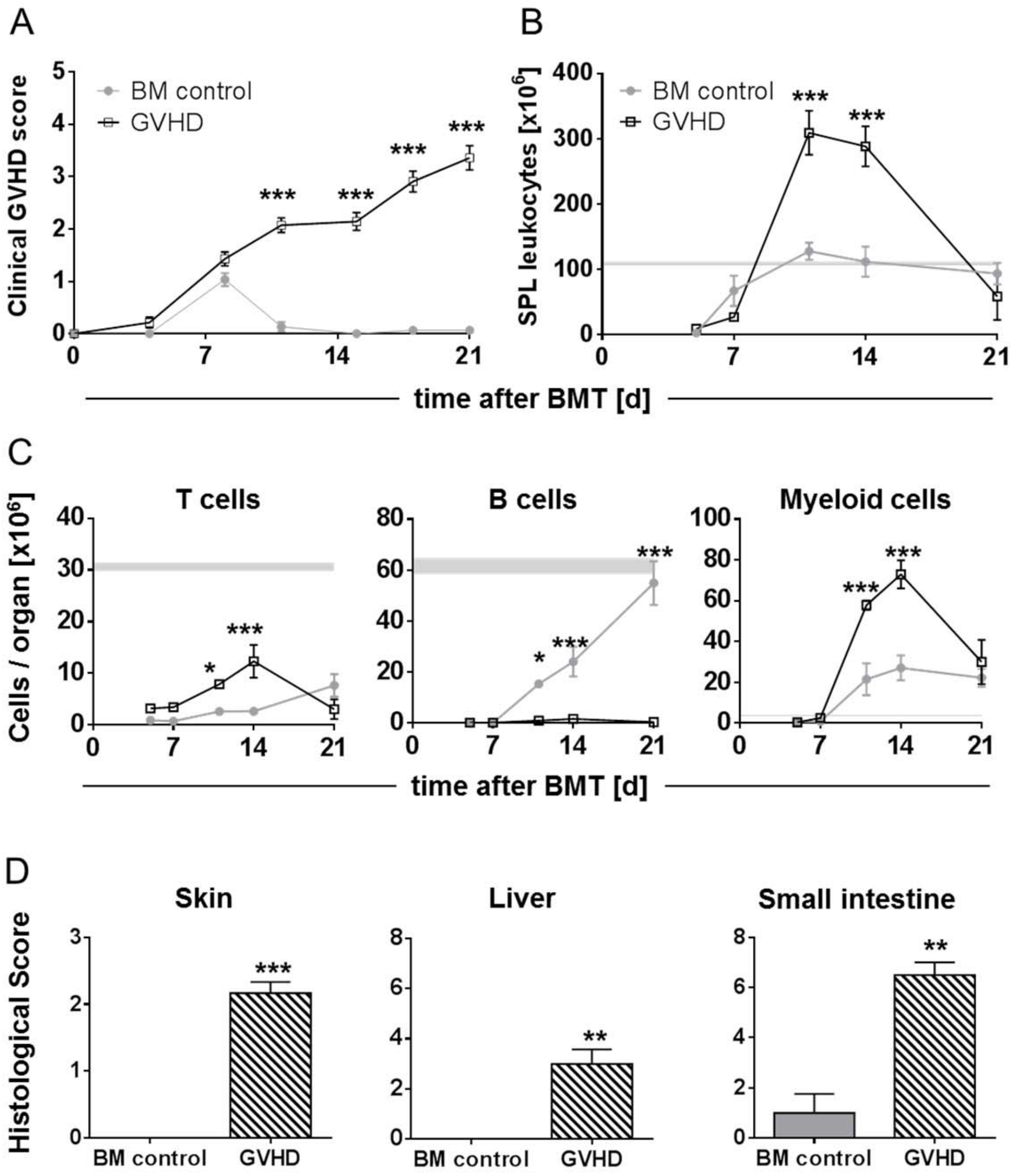

Figure S2: Establishment of d11 after BMT as time point of therapeutic intervention. CB6F1 recipients were lethally irradiated and transplanted with 2.5 x 10^6 BM cells alone (BM control) or with BM and 5 x 10^6 splenocytes (GVHD). (A) Clinical GVHD score. (B) Absolute leukocyte numbers and (C) absolute T cell, B cell and monocyte/granulocyte cell numbers in spleen of transplant recipients at indicated time points. Grey-shaded areas in B and C represent respective cellular levels in non-transplanted CB6F1 mice. (D) Histopathological score of skin, liver and small intestine of transplant recipients on d11 after BMT. Data represent mean ± s.e.m of n=18 (GVHD) and n=21 (BM control) per time point in (A) and n= 3/group and time point in (B)-(D). *p <0.05; **p<0.01; ***p<0.001.

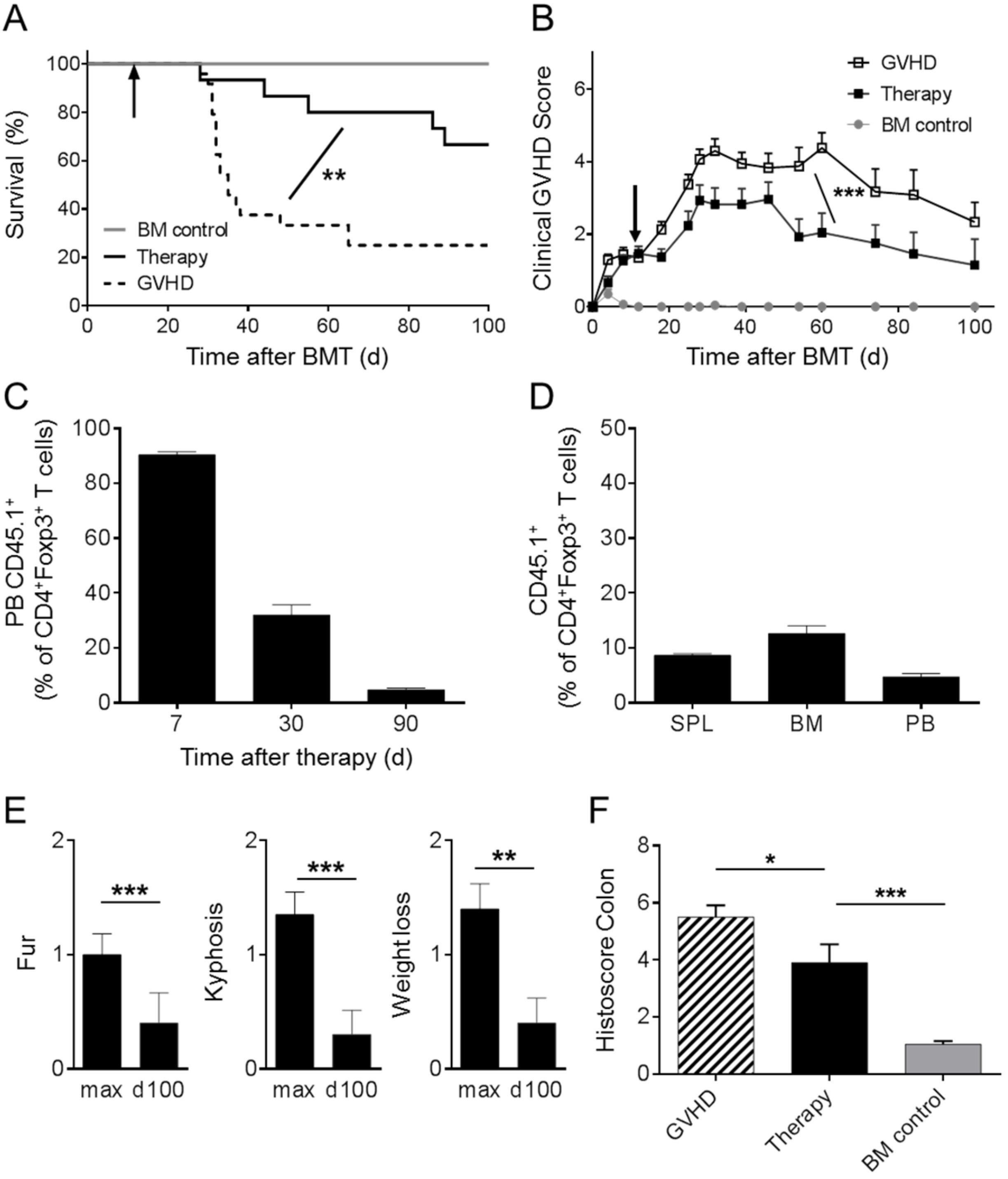

Figure S3: Efficacy of Treg therapy is independent of the donor strain. Lethally irradiated CB6F1 recipients were transplanted with 2.5 x 10^6 C57BL/6 BM cells either alone (BM control) or together with 15 x 10^6 C57BL/6 splenocytes (GVHD). On d11 after BMT part of the GVHD animals received 15 x 10^6 *in vitro* expanded C57BL/6 WT (A,B,E,F) or CD45.1$^+$ (C,D) Treg (Therapy). (A) Survival is shown as combined data from 4 independent experiments with *n*=5 (BM control), *n*=15 (Therapy) and *n*=24 (GVHD) mice/group. Treg application is indicated by the black arrow. Only 25% of untreated mice with GVHD survive the observation period of 100 d, whereas Treg-treated mice show a significantly improved survival (66.7%). (B) Clinical GVHD score of the animals shown in (A). Persistence of therapeutic Treg in PB (C) at indicated time points after therapy and (D) in indicated organs at d90 after therapy (*n*=4-8/time point/organ). (E) Peak and final clinical scores (max. score = 2 per parameter) for fur texture (including alopecia), kyphosis and weight loss of animals surviving for >90 d after Treg therapy (*n*=10; 2 independent experiments). (F) Histopathological colon score. Mice with GVHD (*n*=16) were analyzed on d100 or when moribund; 'Therapy' (*n*=10) and 'BM control' mice (*n*=23) were analyzed on d100 after BMT. Data are from 4 independent experiments. (* $p<0.05$, ** $p<0.01$, *** $p<0.001$).

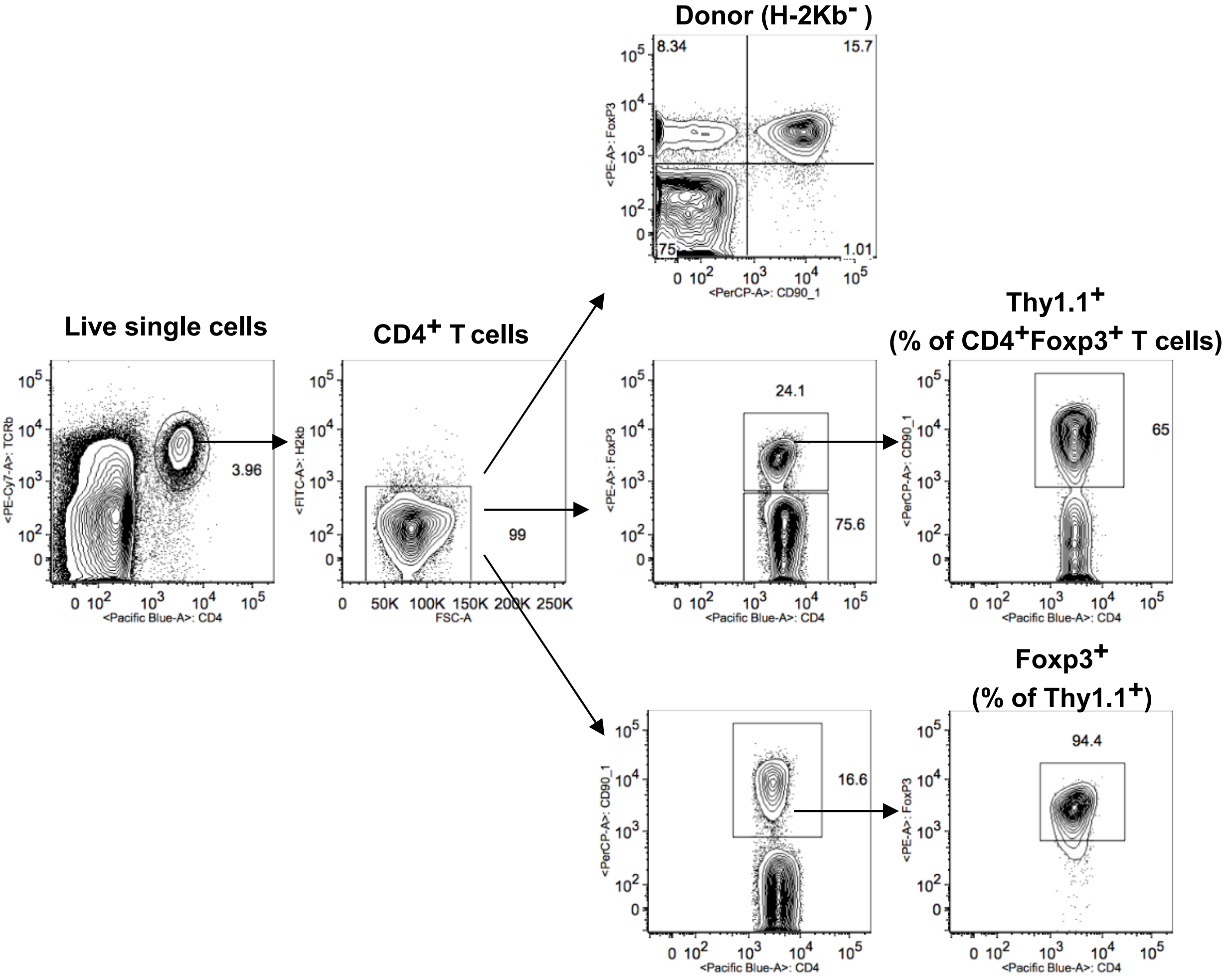

Figure S4 (related to Fig. 3): Identification of therapeutically applied Treg in allo BMT recipients. CB6F1 recipients (H-$2^{d/b}$) of BALB/c WT (H-2^d;Thy1.2^+) BM and splenocytes were analysed 30d after treatment with BALB/c_Thy1.1^+ (CD90.1^+) Treg. Sequential gating strategy is shown to identify all donor CD4 T cells (TCRβ$^+$CD4$^+$H-2Kb$^-$; middle row, first two panels), all donor Treg cells (Foxp3$^+$ subset of donor CD4 T cells; upper row and middle row, third panel), the proportion of therapeutic (Thy1.1^+) Treg among all donor Treg cells (middle row, fourth panel) and the frequency of Foxp3$^+$ cells among all therapeutically applied Thy1.1 (CD90.1)$^+$ Treg cells (lower row, second panel).

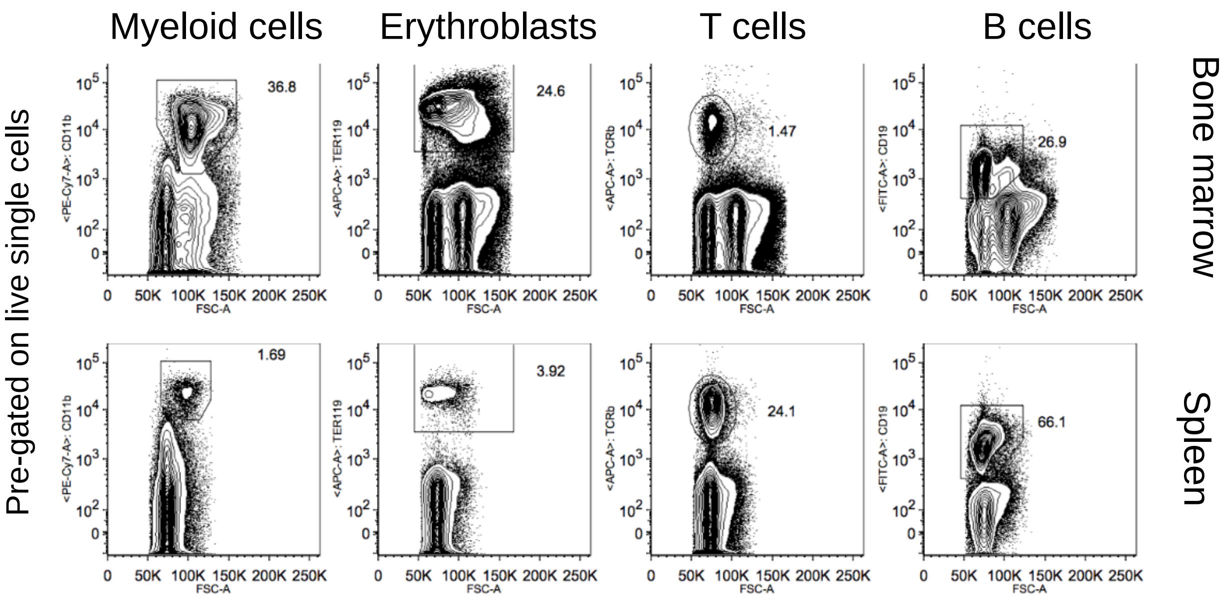

Figure S5 (rel. to Fig.4): Identification of cellular subsets in bone marrow and spleen of allo BMT recipients. Exemplary gating of indicated cellular subsets (CD11b$^+$ myeloid cells, Ter119$^+$ erythroid precursors and erythroblasts, TCRβ$^+$ T cells and CD19$^+$ B cells) in bone marrow and spleen of CB6F1 mice transplanted with BM cells only and analysed on d100 after BMT.

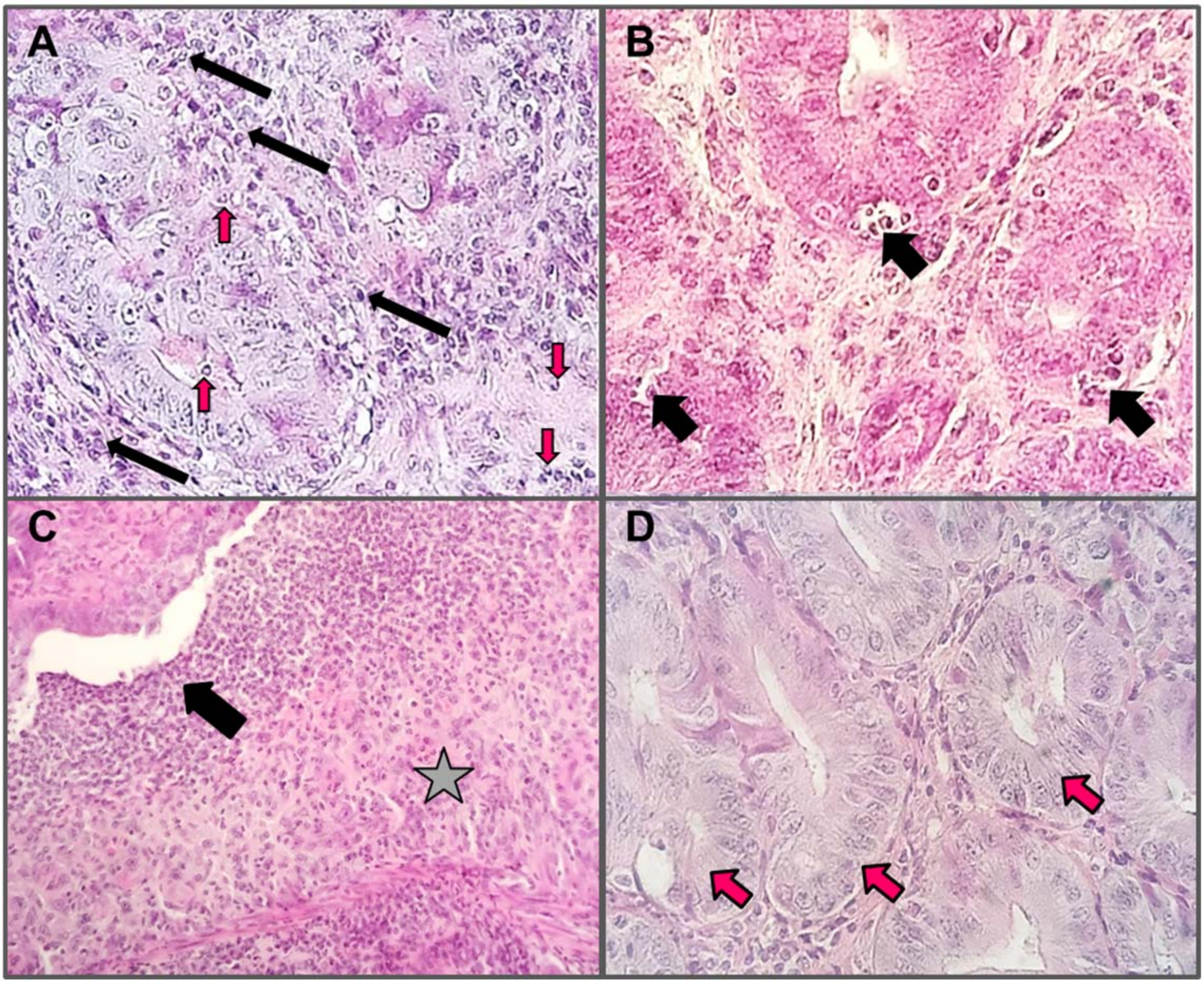

Figure S6: aGVHD in the haploidentical BALB/c → CB6F1 model is associated with severe tissue damage in the GI tract. Representative H/E-stained tissue sections from colonic lamina propria of CB6F1 recipients at d40 after transplantation with bone marrow cells and splenocytes from BALB/c donors. (A) shows a mixed inflammatory infiltrate with lymphocytes (black arrows) and neutrophils (magenta arrows). (B) shows epithelial cell apoptosis (black arrows). (C) depicts deep mucosa ulceration (arrow) and crypt loss (asterisk). (D) shows crypt distorsion (magenta arrows). A, B and D: 400x; C: 200x magnification.
